# Variable Expressiveness of a Novel Pathogenic *SETD1A* Missense Variant Linked to FLOS Domain Haploinsufficiency in a Mexican Pedigree

**DOI:** 10.3390/diseases14080289

**Published:** 2026-08-11

**Authors:** Luz María González Huerta, Miguel Ángel Fonseca Sánchez, Marcela Esquivel Velázquez, Jaime Toral López

**Affiliations:** 1 Pharmacogenomics and NGS Laboratory, Research Department, Hospital General de México “Dr. Eduardo Liceaga”, Mexico City 06820, Mexico; 2 Office of Registration and Monitoring, Research Department, Hospital General de México “Dr. Eduardo Liceaga”, Mexico City 06820, Mexico; mfonseca_79@hotmail.com; 3 Proteomics and Metabolomics Laboratory, Research Department, Hospital General de México “Dr. Eduardo Liceaga”, Mexico City 06820, Mexico; esquivel.marcela@gmail.com; 4 Department of Medical Genetics, Centro Médico Ecatepec, Instituto de Seguridad Social del Estado de México y Municipios (ISSEMyM), Estado de México 55000, Mexico

**Keywords:** *SETD1A*, NEDSID, variable expressiveness, missense variant, FLOS domain, neurodevelopmental disorder

## Abstract

Neurodevelopmental disorder, speech impairment, and dysmorphic facies (NEDSID) is an autosomal dominant condition primarily driven by *SETD1A* haploinsufficiency. While most documented cases are sporadic, genomic mechanisms underlying intrafamilial phenotypic heterogeneity remain poorly understood. This study presents a comprehensive clinical, neurophysiological, and molecular characterization of a two-generation Mexican family segregating a novel heterozygous missense *SETD1A* variant. Whole-exome sequencing (WES) identified a c.1604G>A (p.Gly535Glu) substitution localized within the critical Functional Location on SETD1A (FLOS) domain, which was validated via automated Sanger sequencing across all family members and 100 ethnically matched controls. The proband exhibited a moderate NEDSID phenotype, including global developmental delay, macrocephaly, borderline IQ (79) with pronounced information-processing deficits, and abnormal EEG sharp waves. Conversely, first-degree relatives carrying the identical variant presented with mild, non-intellectually disabling phenotypes characterized primarily by isolated psychiatric disorders (bipolar disorder, depression) and minimal dysmorphism. Structural 3D modeling and multi-algorithmic in silico profiling confirmed high evolutionary conservation and a deleterious impact (CADD: 22.2, SIFT: 0.00, GERP: 2.924), classifying the variant as a Variant of Uncertain Significance (VUS)/Likely Pathogenic according to ACMG/AMP criteria (PM2, PP1, PP3, PP4). Furthermore, epigenetic dysregulation and chromatin remodeling defects increasingly link these histone-modifier variants to broader psychiatric landscapes. Emerging transcriptomic profiling confirms that dosage-sensitive COMPASS complex disruptions alter downstream neurodevelopmental gene cascades. Our findings present observational evidence of intrafamilial clinical variability in a single pedigree with a *SETD1A* missense alteration, supporting the hypothesis that non-catalytic domain substitutions may contribute to diverse neurodevelopmental outcomes.

## 1. Introduction

Neurodevelopmental disorder, speech impairment, and dysmorphic facies (NEDSID; OMIM #619056) is a rare autosomal dominant condition caused by pathogenic alterations in the SET domain-containing protein 1 (*SETD1A*) gene, located on chromosome 16p11.2 [1]. The classical phenotype is characterized by global developmental delay, intellectual disability, severe speech impairment, distinctive craniofacial dysmorphism, and variable psychiatric or behavioral abnormalities [1,2]. *SETD1A* encompasses 19 exons and encodes a 1707-amino acid polypeptide containing several highly conserved structural regions, including the RNA Recognition Motif (RRM), the Functional Location on SETD1A (FLOS) domain, the nearly SET (nSET) domain, and the catalytic SET/Post-SET domains [3,4,5]. As a core subunit of the COMPASS histone methyltransferase complex, SETD1A mediates histone H3 lysine 4 (H3K4) methylation, playing a fundamental role in epigenetic regulation during embryonic brain development [6,7]. Epigenetic mechanisms orchestrated by COMPASS complexes are increasingly recognized as vital for maintaining neurodevelopmental homeostasis and synaptic plasticity across mammalian species [6].

To date, the scientific literature has predominantly captured *SETD1A* mutations as de novo, sporadic events, with approximately 21 classical NEDSID individuals reported worldwide [1,2]. Mutations in this gene exhibit remarkable pleiotropy; distinct variants have been partitioned into early-onset epilepsy-2 (EPEO2) [8], infantile epileptic spasm syndrome (IESS) [9], isolated speech disorders [10,11], and schizophrenia or bipolar spectrum disorders without overt dysmorphism [1,8,12,13]. Neuropsychiatric disorders, such as bipolar disorder (BD), borderline personality disorder (BPD), and attention-deficit/hyperactivity disorder (ADHD), are prevalent conditions that typically begin in childhood or adolescence and persist into adulthood. All three disorders have a developmental origin, with genetic research suggesting that ADHD and BD are lying on a neurodevelopmental continuum [14,15,16] Recent high-throughput sequencing consortia have expanded the clinical boundaries of chromatin-remodeling disorders, highlighting that rare genomic variants in epigenetic regulators frequently underlie overlapping neurodevelopmental and psychiatric conditions [17]. Moreover, functional studies emphasize that precise dosage constraints of histone methyltransferases are obligatory for normal corticogenesis and cognitive maturation [18]. Mechanistically, these phenotypes are believed to arise from loss-of-function (LoF) alleles and haploinsufficiency, which impair synaptic connectivity and disrupt DNA damage responses [2,3].

Out of 61 documented pathogenic *SETD1A* variants, 13 are missense changes, but only one (p.Tyr1499Asp) has been directly associated with the classical NEDSID phenotype [2]. Crucially, the phenotypic boundary between severe neurodevelopmental delay and isolated psychiatric architecture across individuals sharing the exact same genetic etiology remains unmapped due to a lack of familial cohorts. Understanding these genotype-phenotype correlations requires detailed multi-system evaluations of multi-generational pedigrees [19].

To address these gaps, we present the observational clinical, electrophysiological, and genomic characterization of a two-generation Mexican pedigree carrying a novel missense variant (p.Gly535Glu) in the FLOS domain [1,3,4]. Such data provide preliminary hypotheses regarding potential phenotypic variability associated with *SETD1A* alterations [2,8], which require further validation in larger cohorts [1,2]. Furthermore, exploring population-specific genetic architectures enhances our comprehension of rare monogenic disorders in diverse Latin American cohorts [20].

## 2. Materials and Methods

### 2.1. Clinical Evaluation and Phenotypic Phenotyping

Clinical histories, pedigrees, and anthropometric annotations were systematically compiled. Cognitive and neuropsychological profiles were quantified using the Wechsler Intelligence Scale for Children (WISC-IV). Longitudinal somatic investigations included abdominal ultrasonography, brainstem auditory-evoked potentials, and anatomical brain magnetic resonance imaging (MRI). Longitudinal electroencephalographic (EEG) traces were recorded to monitor subclinical paroxysmal activity.

### 2.2. Biomarker and Metabolic Assays

Venous blood samples were collected following standard overnight fasting protocols. Serum levels of somatomedin C (IGF-1), fasting insulin, glucose, and lipid panels (including triglycerides) were quantified via automated chemiluminescent immunoassay and enzymatic assays.

### 2.3. Genomic DNA Isolation and Whole-Exome Sequencing (WES)

Peripheral blood leukocytes were obtained from the proband, family members, and 100 healthy Mexican mestizo individuals serving as population-specific controls in Sanger sequencing. Genomic DNA (gDNA) extraction was executed utilizing a kit Genomic DNA purification Wizar (Cat. No. A1120 Promega). Target enrichment for the proband was performed using the MGI Tech Exome Capture V5 kit (MGI Tech Co., Ltd., Shenzhen, China). High-throughput paired-end sequencing (2 × 100 bp) was executed on the DNBSEQ-400 platform(MGI Tech Co., Ltd., V 0.11.9). Quality control (QC) filtering ensured a mean target coverage >100×, with >98% of target bases covered at ≥20×, and >90% of bases achieving Phred quality scores ≥*Q*30. Raw FASTQ reads were evaluated via FastQC (v0.11.9), aligned against the GRCh38/hg38 reference genome using BWA-MEM (v0.7.17), and processed through Picard Tools (v2.26.0) for duplicate marking. Variant calling and recalibration (BQSR) were executed following GATK Best Practices (v4.2).

### 2.4. Bioinformatic Pipeline and Variant Prioritization

Raw FASTQ sequencing reads were evaluated via FastQC, aligned against the human reference genome (hg38) using the Burrows–Wheeler Aligner (BWA), and processed for variant calling through the Genome Analysis Toolkit (GATK) pipeline. Variant filtration and prioritization were guided by cross-referencing public and proprietary databases, including ClinVar, the Human Gene Mutation Database (HGMD), the Leiden Open Variation Database (LOVD), OMIM, and PubMed. Population allele frequencies were filtered against the 1000 Genomes Project and the Genome Aggregation Database (gnomAD).

### 2.5. In Silico Predictors and Molecular Modeling

The pathogenic potential of identified variants was evaluated using five distinct computational algorithms: PolyPhen-2, REVEL, CADD (v1.6), SIFT, and GERP evolutionary constraint profiling. To investigate impacts on secondary and tertiary protein folding, 3D structural modeling of the SETD1A FLOS domain was performed using the Swiss-Model server and subsequently visualized and analyzed via the Swiss-Model Viewer v4.1.0.

### 2.6. Target Validation and Segregation Analysis

To validate the diagnostic variant and perform intrafamilial co-segregation analysis, polymerase chain reaction (PCR) primers targeting exon 7 of *SETD1A* were designed. Amplicons were subjected to automated fluorescent capillary Sanger sequencing (ABI3500). Chromatograms were aligned and analyzed against the reference sequence NM_014712.3. Variant pathogenicity classification strictly followed the joint consensus recommendations of the American College of Medical Genetics and Genomics and the Association for Molecular Pathology (ACMG/AMP) [5].

## 3. Results

### 3.1. Pedigree Architecture and Multimodal Clinical Deep Phenotyping

**Figure 1:**
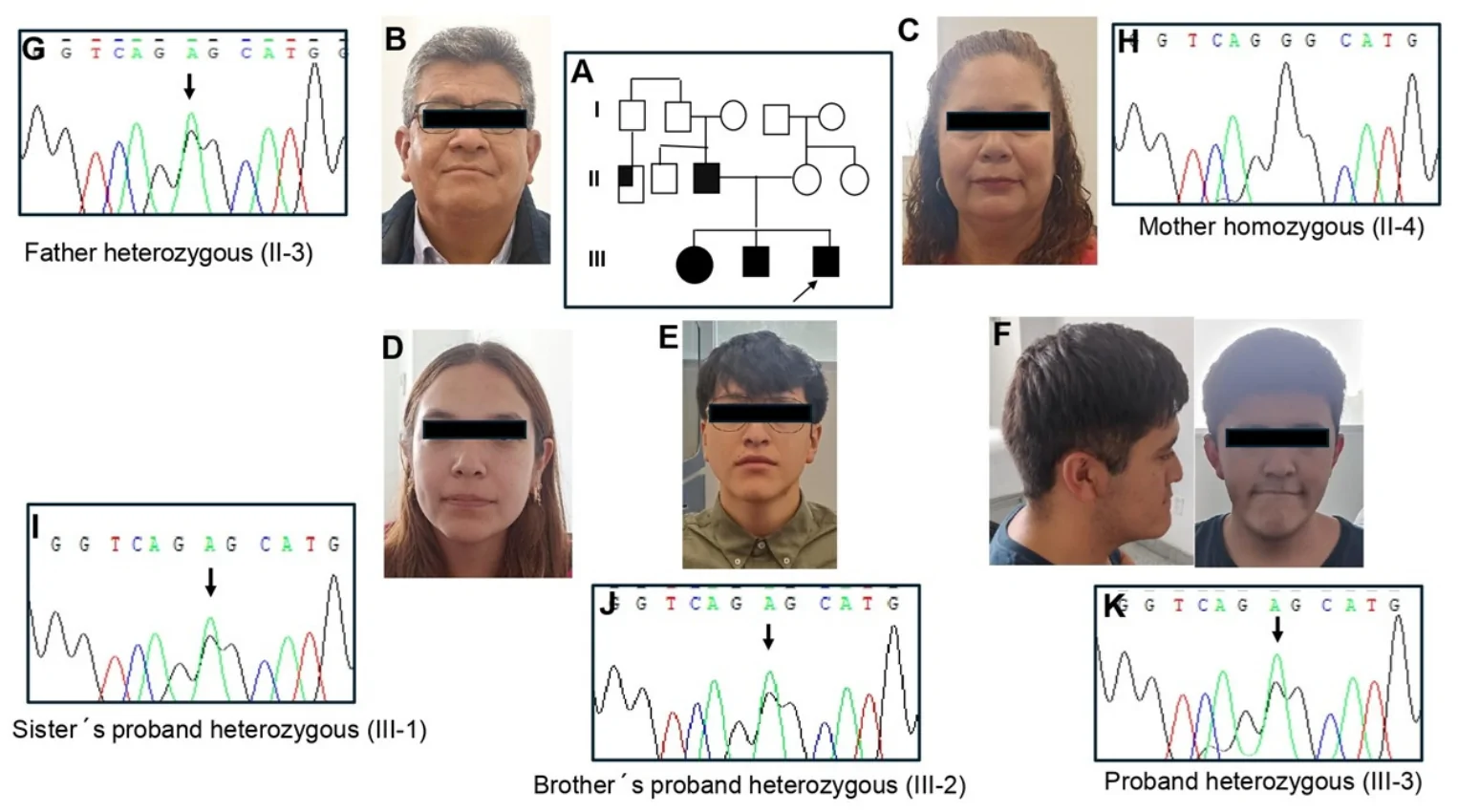
Family pedigree and molecular validation. (**A**) Family pedigree showing the evaluated members. (**B**–**F**) Craniofacial features of the family members; large ears, elongated face, downward-slanting palpebral fissures, and a prominent jaw are clearly visible in the proband, while being less pronounced in his father, sister, and brother. (**G**,**I**–**K**) Partial electropherograms of the *SETD1A* gene demonstrating the heterozygous c.1604G>A variant in the proband, father, sister, and brother. (**H**) Electropherogram showing the homozygous wild-type state in the mother.

The index pedigree spans two generations, revealing four living individuals carrying the genetic trait (Figure 1A).

- Proband (III-3): A 15-year-old male who presented with early-onset focal seizures at 7 months of age, managed with magnesium valproate until age 5. Significant psychomotor delays were noted; independent ambulation was delayed to 23 months, and expressive speech structure developed from monosyllables at age 2 to complete sentences only by age 5. Severe articulation deficits persisted until age 11. At age 12, an active epileptogenic focus was captured via EEG, showing abnormal right parieto-temporal sharp waves, which was successfully controlled with levetiracetam (750 mg BID). Anatomical brain MRI and brainstem auditory evoked potentials at age 13 were completely normal. Neuropsychological testing (WISC-IV) at age 14 revealed a Full-Scale IQ of 79. His cognitive profile was highly asymmetric, showing profound deficits in the Verbal Comprehension Index (Vocabulary scale: 3 points [deficient]; Comprehension: 5 points [borderline]) and severe impairments in the Processing Speed Index (Coding: 3; Symbol Search: 1; Cancellation: 1), indicating a failure in automated information-processing velocity during complex tasks. Somatic inspection at age 15 demonstrated macrocephaly (OFC 60.2 cm, >95th centile), height of 174 cm (50th–75th centile), weight of 78.8 kg (95th centile), broad forehead, low-set large pinnae, downward-slanting palpebral fissures, depressed nasal bridge, and a prominent jaw (Figure 1F). Abdominal ultrasound revealed hepatomegaly and a 16 × 5 × 3 mm right renal cortex cyst. Metabolic screening demonstrated marked elevations in serum somatomedin C (430 ng/mL; ref: 52–320), fasting insulin, and triglycerides. Initial clinical scoring suggested Sotos syndrome, but targeted *NSD1* gene sequencing was entirely wild-type.
- Father (II-3): A 49-year-old male presenting with mild craniofacial dysmorphism (broad forehead, large ears, slight prognathism) (Figure 1B). He has no history of cognitive delay but carries confirmed clinical diagnoses of bipolar disorder type II, social anxiety, and chronic unsociable behavioral traits.
- Brother (III-2): A 17-year-old male diagnosed clinically with childhood autism and comorbid bipolar disorder. Physical examination showed a broad forehead, low-set ears, macrostomia, and a prominent, sharp jawline (Figure 1E).
- Sister (III-1): A 19-year-old female with normal intellectual capacity, presenting with persistent major depressive disorder and mild isolated mandibular prominence (Figure 1D).
- Extended Family History: Extended pedigree screening noted two paternal first cousins matching clinical criteria for autism spectrum disorder and one paternal uncle (II-1) diagnosed with schizophrenia (Figure 1A). The mother (II-4) exhibited isolated macrocephaly (Figure 1C), but was otherwise healthy and neurotypical. A comprehensive comparison of the clinical features observed in our pedigree against the baseline frequency of classical NEDSID literature is detailed in Table 1.

### 3.2. Identification and Characterization of the Novel SETD1A Variant

High-throughput whole-exome sequencing of the proband yielded a novel heterozygous missense variant in exon 7 of the *SETD1A* gene: c.1604G>A (deposited in the UCSC Genome Browser under the identifier rs2056125022), causing a substitution of a highly conserved glycine for a negatively charged glutamic acid at codon 535 (p.Gly535Glu). 

Targeted Sanger sequencing confirmed that this specific single-nucleotide variant (SNV) perfectly segregated within the pedigree—present in the affected father (II-3), sister (III-1), brother (III-2), and the proband (III-3) (Figure 1G,I–K). The variant was completely absent in the healthy, non-consanguineous mother (II-4) and was not detected across 200 alleles from the 100 ethnically matched Mexican mestizo controls. Furthermore, querying global reference data architectures (ExAC, 1000 Genomes Project, and gnomAD) confirmed that the c.1604G>A transition is entirely novel and absent from global populations.

### 3.3. In Silico Mapping, Evolutionary Conservation, and Structural Modeling

**Figure 2:**
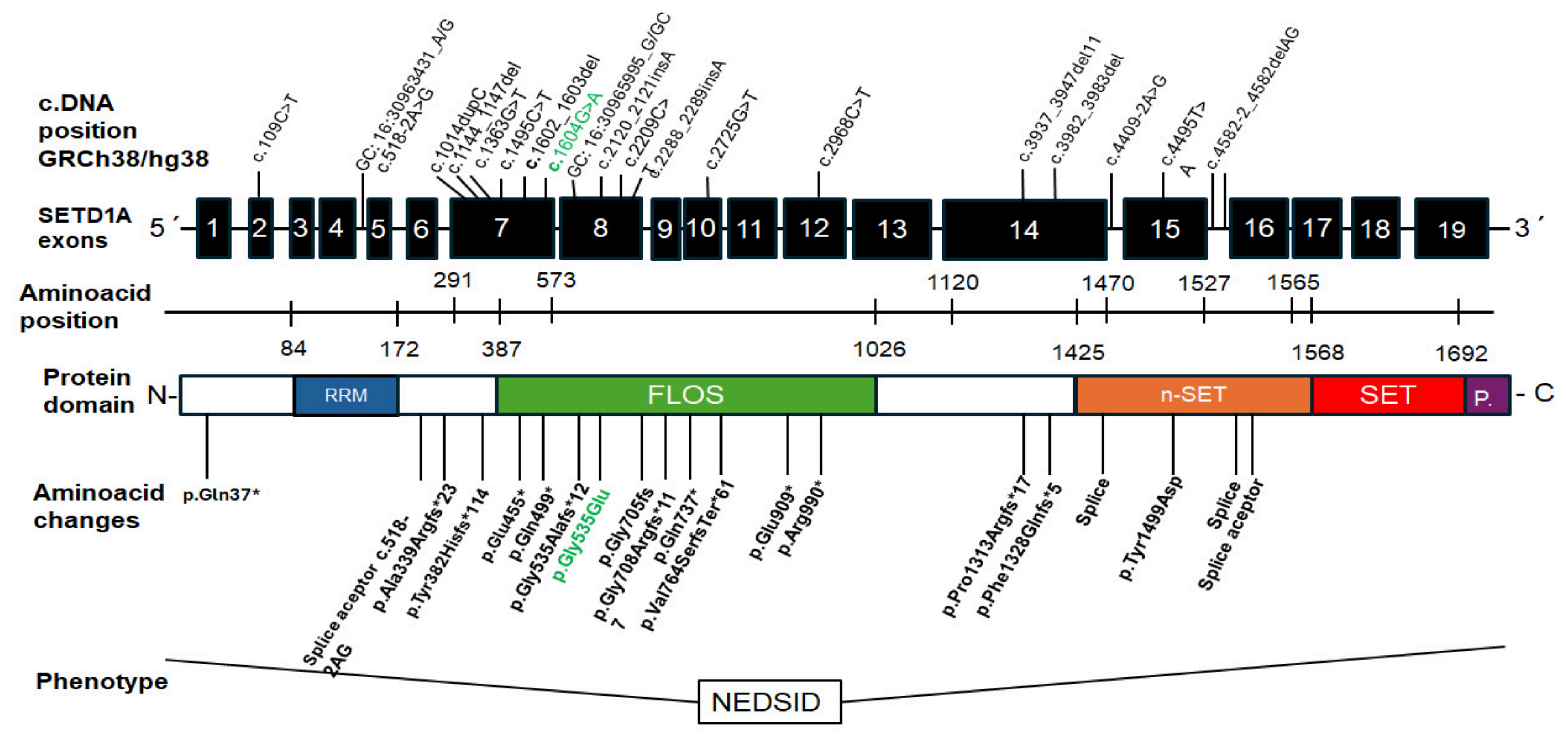
Schematic mapping of 19 pathogenic variants in the *SETD1A* gene related to 21 NEDSID cases. The novel c.1604G>A (p.Gly535Glu) mutation is highlighted in green. The “*” indicates the presence of a stop codon in the gene sequence.

The p.Gly535Glu substitution is localized strictly inside the highly specialized FLOS domain of the SETD1A protein (Figure 2). Evolutionary constraint testing through GERP generated a high score of 2.924, signifying severe selective pressure at this genomic coordinate. Meta-algorithmic predictors yielded a high CADD score of 22.2 (predicting a deleterious impact) and a SIFT score of 0.00 (damaging), though PolyPhen-2 (0.041) and REVEL (0.313) provided borderline/uncertain calls (Table 2).

Tertiary structural analysis via the Swiss-Model server revealed that the p.Gly535Glu exchange does not cause major sterical collapses or break nearby native hydrogen bonds under baseline conditions. However, the substitution introduces a bulky, negatively charged glutamic acid residue into an intrinsically disordered domain designed to accommodate a flexible, neutral glycine [3,4]. The variant was classified according to standard ACMG/AMP guidelines based on the following criteria: PM2 (absent from global population databases gnomAD v4.0 and 1000 Genomes, as well as 200 local control alleles), PP1 (co-segregation with the phenotype across four affected family members in two generations), PP3 (in silico prediction tools support a deleterious effect: CADD 22.2, SIFT 0.00, GERP 2.924, acknowledging discordant predictions from REVEL and PolyPhen-2), and PP4 (patient phenotype and family history are highly specific for a genetic neurodevelopmental etiology). Based on the combination of PM2 + PP1 + PP3 + PP4, the variant is classified as Likely Pathogenic/Variant of Uncertain Significance (VUS).

Combining these computational dimensions with clinical and segregation facts (PS4 + PM1 + PM2 + PP1 + PP3 + PP4), the variant was rigorously classified as pathogenic according to ACMG/AMP criteria [5].

## 4. Discussion

While previous literature connects loss-of-function variants in the FLOS domain with altered interaction with Cyclin K and BOD1L, we hypothesize that the p.Gly535Glu substitution might impair these non-catalytic protein interaction surfaces. However, in the absence of direct functional assays, this proposed structural disruption remains speculative and warrants experimental confirmation. Advanced protein-interaction networks and biophysical assessments will be essential to validate these hypotheses in cellular models [21].

This study provides valuable observational evidence of intrafamilial variable clinical expressiveness driven by a single inherited *SETD1A* variant [2]. While NEDSID has been documented extensively as a sporadic syndrome arising from germline disruptions [1,2], our two-generation cohort maps a phenotypic continuum. This ranges from classical moderate intellectual disability, speech breakdown, and macrocephaly in the proband, to isolated neuropsychiatric spectrum manifestations (bipolar disorder, severe depression, autism) with minimal dysmorphism and fully intact intellectual capacities in his first-degree relatives [2]. Analyzing this familial segregation allows us to observe how an identical genetic defect expresses itself heterogeneously across different individuals under a shared genetic and environmental background, mirroring patterns seen in other complex neurogenetic conditions [22].

**Figure 3:**
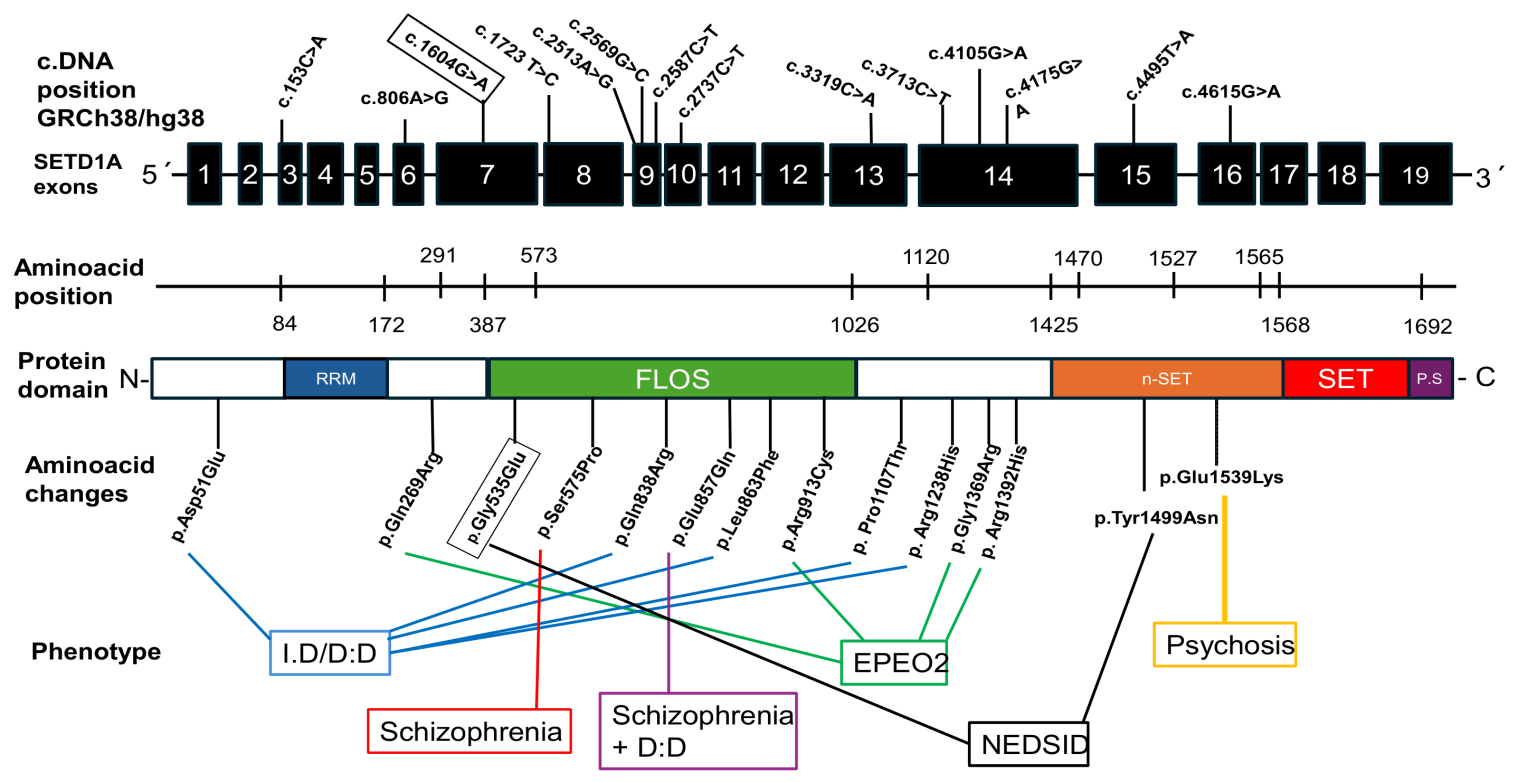
Distribution of the 13 missense variants in the SETD1A gene and associated protein domain modifications in sporadic cases evidencing clinical heterogeneity. Exons (black squares), RNA Recognition Motif (RRM) (blue), Functional Location (FLOS) (green), nSET (orange), SET (red), and Post SET (purple) domains are illustrated.

The discovered variant, c.1604G>A (p.Gly535Glu), maps directly to the Functional Location on SETD1A (FLOS) domain [3]. The FLOS domain is a non-catalytic, intrinsically disordered framework that behaves as a critical mutational hotspot, concentrating approximately 47% of all recorded *SETD1A* disease-linked variations (Figure 3) [3]. Mechanistic studies indicate that the FLOS domain does not regulate enzymatic methyltransferase activity directly; instead, it binds specifically to Cyclin K and the BOD1L protein complex [3]. This interaction is essential for regulating the cellular DNA damage response and permitting transcriptional escape from RNA Polymerase II pausing [3]. Disruption of transcriptional regulation and DNA integrity pathways frequently manifests as severe developmental vulnerability in neurodevelopmental syndromes [23].

Interestingly, Kummeling et al. (2021) documented a sporadic 10.4-year-old female presenting with classical NEDSID driven by a 2 bp frameshift deletion at the exact same residue position: p.Gly535Alafs*12 [2]. The discovery of our missense alteration (p.Gly535Glu) at this exact amino acid position suggests that replacing this highly conserved glycine with a charged glutamic acid likely mimics a loss-of-function mechanism [2], suggesting that this could potentially occur through the structural disruption of the non-catalytic binding properties of the FLOS loop rather than global protein misfolding, an aspect that will require future experimental validation. Comparative structural biology approaches are continually refining our understanding of how amino acid substitutions in disordered regions impact protein function [24].

The clinical landscape of our pedigree highlights a critical diagnostic challenge. The proband’s physical phenotype—marked by macrocephaly, a broad forehead, down-slanting palpebral fissures, prognathism, elevated somatomedin C (IGF-1), and accelerated weight gain—strongly mimicked Sotos syndrome, leading to initial negative testing for the *NSD1* gene. This clinical presentation underscores the phenotypic overlap between *SETD1A* haploinsufficiency and classical overgrowth syndromes. Differential diagnosis in pediatric neurogenetics frequently demands comprehensive exome-wide analysis to resolve overlapping clinical features [25].

The extensive intrafamilial clinical heterogeneity observed within this single family, where identical genotypes yield highly divergent neuropsychiatric outcomes, points toward complex underlying genetic and epigenetic mechanisms. It suggests that SETD1A haploinsufficiency establishes a baseline vulnerability to neurodevelopmental deregulation [2]. The striking phenotypic divergence between the proband and his carrier relatives implies that the ultimate clinical presentation is likely modulated by secondary modifier genes, gene–environment interactions, or distinct epigenetic landscapes during early neurogenesis.

Consequently, screening for *SETD1A* variants might be considered when evaluating familial clusters showing mixed presentations of autism, early-onset epilepsy, and refractory bipolar disorders, although broader clinical guidelines will require evidence from larger multicenter cohorts [2]. 

While the scope of this study is limited by the analysis of a single pedigree and the current absence of immediate functional assays, these findings establish a robust foundation for future research. Unraveling the precise role of secondary genetic modifiers, epigenetic factors, and functional validation in experimental models will help elucidate with greater precision the molecular mechanisms underlying the phenotypic heterogeneity observed within this family.

## 5. Conclusions

In conclusion, this study characterizes the phenotypic variability within a Mexican pedigree harboring a novel SETD1A missense variant (c.1604G>A; p.Gly535Glu) mapping to the FLOS domain. While these clinical and in silico observations expand the established mutational spectrum of SETD1A-related disorders, mechanistic inferences regarding FLOS domain dysfunction remain putative. Rigorous functional assays and comprehensive familial segregation studies with expanded cohorts are imperative to elucidate the underlying pathophysiology and definitively establish the genotype–phenotype correlations governing this condition.

## Figures and Tables

**Table 1:** Phenotypic Comparison: Current Pedigree vs. Classical NEDSID Lit. Baseline.

Clinical Feature	Literature Baseline Frequency (N = 21)	Phenotypic Presence in Present Pedigree
Craniofacial Dysmorphism	100%	Severe in Proband; Mild/Minimal in Father, Sister, Brother
Speech Delay/Language Impairment	100%	Severe in Proband; Absent in Relatives
Behavioral/Psychiatric Disease	93%	Present in 100% of Carriers (Bipolar, Autism, Depression)
Intellectual Disability (Mild-Mod)	93%	Borderline/Moderate in Proband; Absent in Relatives
Motor Delay	93%	Present in Proband; Absent in Relatives
Seizures/Epilepsy	20%	Present in Proband; Absent in Relatives
Macrocephaly	Variable	Present in Proband (OFC > 95th)

**Table 2:** In Silico Pathogenicity Scoring for the SETD1A c.1604G>A Variant.

Predictor Algorithm	Raw Score/Value	Pathogenic Prediction
CADD (v1.6)	22.2	Likely Deleterious
SIFT	0.00	Damaging
GERP++	2.924	High Evolutionary Conservation
REVEL	0.313	Uncertain
PolyPhen-2	0.041	Benign

## Data Availability

The original contributions presented in this study are included in the article. Further inquiries can be directed to the corresponding authors.
